# Inhibitory Effect of Transfer Factor on Avian Reticuloendotheliosis Virus Infection in Chicks

**DOI:** 10.3390/vetsci12111041

**Published:** 2025-10-31

**Authors:** Xinli Wang, Mengyu Song, Qingyue Wu, Zhihao Ren, Wenping Cui, Yixin Wang, Shuang Chang, Peng Zhao

**Affiliations:** 1 College of Veterinary Medicine, Shandong Agricultural University, Tai’an 271018, China; wangxinli0814@163.com (X.W.); smyu110@163.com (M.S.); wuqingyue0315@163.com (Q.W.); renzhihao@sdau.edu.cn (Z.R.); sducwp@163.com (W.C.); 07sdauwyx@163.com (Y.W.); changshuang81@126.com (S.C.); 2 Shandong Provincial Key Laboratory of Animal Biotechnology and Disease Control and Prevention, Tai’an 271018, China

**Keywords:** avian reticuloendotheliosis virus, transfer factor, immunosuppression, vertical transmission, horizontal transmission

## Abstract

**Simple Summary:**

Reticuloendotheliosis virus (REV) infects chicks through multiple routes, causing the disease reticuloendotheliosis. This disease slows chicks’ growth, damages their organs, and even leads to death, and there is still no commercial vaccine to prevent it. To find a way to control this virus, this study tested a substance called transfer factor (TF) in three scenarios: using it on chicks before they were infected, on 1-day-old chicks after infection, and on chicks infected during the incubation period. The results showed that transfer factor improved the chicks’ growth, reduced organ damage and mortality, and also decreased the amount of virus in their bodies and inhibited cloacal virus shedding. Using more transfer factor or using it more frequently enhanced its effectiveness. This finding helps reduce the harm of reticuloendotheliosis to poultry farming, protects chick health, and ensures stable chicken production.

**Abstract:**

Reticuloendotheliosis virus (REV) has multiple transmission routes and can induce severe immunosuppression upon infection. Three application scenarios were set up to explore transfer factor’s (TF’s) potential in controlling REV. These scenarios involved using TF in chicks infected with REV during incubation, in 1-day-old chicks post-infection, and in chicks prior to REV infection. The application value of TF in controlling REV was evaluated based on various indices, including weight gain, organ development, and virus replication. The results all indicate that the use of TF could effectively alleviate the developmental delay, hepatomegaly, and thymic atrophy caused by REV infection through different routes, as well as significantly reduce the mortality rate. It could also effectively inhibit REV replication in vivo and cloacal virus shedding. Notably, TF had apparent advantages in inhibiting cloacal virus shedding through the egg in the chicks infected with REV. The functions of TF are dose-dependent, where increasing the dose or frequency of use is beneficial for enhancing its effect. This study observed the role of TF in controlling REV infection under different application scenarios, providing necessary auxiliary means to reduce the harm of REV infection.

## 1. Introduction

As a cornerstone of global animal protein supply, the poultry industry grapples with persistent threats from immunosuppressive viral diseases—among which reticuloendotheliosis virus (REV) stands out for its damaging impact on avian health and production. Classified under the Retroviridae family, REV spreads via both horizontal (direct contact) and vertical (contaminated eggs) routes, triggering immunosuppression, growth retardation, and irreversible damage to key immune organs like the thymus, spleen, and bursa of Fabricius in chickens, turkeys, and other avian species [1]. Compounding the challenge, no commercial vaccines or specific antiviral drugs exist for REV control; current reliance on costly flock purification strategies leaves a critical gap for safe, accessible interventions to curb its economic toll. Transfer factor (TF) is a dialyzable substance released by immunoreactive T lymphocytes that can transfer pathogen-specific sensitization-related immune information. It can specifically transfer some cellular immune functions of donors to recipients without or lacking certain cellular immune functions [2,3,4,5]. Transfer factor (TF) is a new type of immune preparation that can regulate B cells through T cells to improve the host’s humoral immune functions, thus enhancing the body’s ability to resist diseases [6,7]. In avian species, TF exerts targeted effects on the immune system: it can promote the proliferation of peripheral blood T lymphocytes in SPF chickens [8], and chicken-spleen-specific TF has also been shown to enhance immune function in chicks by activating T lymphocytes [6]; it can also upregulate the secretion of antiviral cytokines like interleukin-2 (IL-2) and interferon-γ (IFN-γ) [9]. Moreover, chicken-spleen-derived TF strengthens intestinal mucosal immunity—by increasing small intestinal villus height and goblet cell density, while reducing pro-inflammatory cytokine IL-1 and boosting anti-inflammatory cytokine IL-10 [9]—matching avians’reliance on mucosal defense against viral infections. Notably, TF acts as a supplementary antiviral adjuvant in avian vaccines: when co-administered with Newcastle disease vaccines, it elevates antibody titers, extends immune protection, and reduces viral shedding by enhancing CTL activity and NK cell function [10]; combining TF with CpG ODNs also increases peripheral blood CD8^+^ CTLs by 18–22% to overcome virus-induced immune tolerance [9,10]—a key advantage for REV, which evades host immunity via T cell suppression [11,12]. At present, it is mainly used for the prevention and treatment of viral and bacterial diseases that cannot be controlled by antibiotics or immunoglobulin. It has achieved good results in the prevention and treatment of chicken infectious laryngitis and chicken infectious anemia [8], but the prevention and treatment effects against REV has not yet been reported. Therefore, this study investigated the inhibitory effect of TF on REV infection after different modes to provide effective auxiliary measures for the clinical prevention and control of REV.

This study aimed to confirm whether transfer factor (TF) is effective in alleviating reticuloendotheliosis virus (REV)-induced pathogenesis in specific-pathogen-free (SPF) chicks and to explore differences in its efficacy based on administration dose and REV infection route. Three models simulating real poultry production scenarios were established: TF given before REV infection (preventive use), TF given after REV infection in 1-day-old chicks (for horizontal transmission control), and TF given to chicks congenitally infected with REV via the embryonic yolk sac (for vertical transmission intervention). The key outcomes measured included chick growth performance, immune organ development indices, and viral loads in blood and cloacal swabs. The findings are expected to clarify TF’s efficacy against REV across major transmission routes, providing a practical tool to complement existing flock purification strategies and reduce REV-related losses.

## 2. Materials and Methods

### 2.1. Strains and Reagents

The REV-LN1201 strain was isolated from the plasma of chickens suspected to be infected with avian tumor disease from a parent broiler farm in 2012. The whole genome sequence (GenBank accession number: KU641115.1) was completed, and no other pathogens were identified. The TF was produced and supplied by Shandong Sinder Technology Co., Ltd. (Qingdao, China), with a polypeptide content ≥ 1 μg and nucleotide content ≥ 30 μg per ml in D-ribose per mL oral solution. The TF oral solution used was a commercial finished product with batch number 202401201; its endotoxin level was <0.1 EU/mL (detected by the limulus reagent method, referring to GB/T 14233.2-2005) [13], the polypeptide/nucleotide content decreased by <5% after 6 months of storage at 4 °C (as detected by high-performance liquid chromatography), and a bioactivity pre-experiment showed that TF treatment increased the proliferation rate of peripheral blood T cells in SPF chickens by 25% (MTT method, *n* = 3); all experiments used TF from this single batch to avoid batch-to-batch variations.

### 2.2. Experimental Design for Preventive Effect of TF on REV Infection

A total of 80 1-day-old specific-pathogen-free (SPF) chicks were randomly assigned into four groups of 20 chicks each. The control group was intraperitoneally inoculated with normal saline at 6 days old, and the other three infected groups were intraperitoneally injected with LN 1201 virus solution at 6 days old, with 3000 50% Tissue Culture Infectious Dose (TCID_50_)/chicken. The TF was previously administered orally for 3 consecutive days at 2–4 days old, with the low- and high-dose TF groups receiving 0.25 and 0.5 mL/chicken per day, respectively; the TF was not administered in the infection control group. All the chickens were weighed at 7, 14, 21, and 28 days old, and anticoagulated blood and cloacal cotton swabs were collected to detect the REV viral load. At 28 days old, all the chickens were dissected, and the development index of immune organs was measured. In commercial poultry farming, REV commonly infects chicks after 1 week of age. Early TF administration is designed to establish preliminary immune enhancement during the susceptible period, matching the practical ‘prevention-oriented’ demand for REV control.

### 2.3. Inhibitory Effect of TF on REV Horizontal Propagation

A total of 80 1-day-old SPF chicks were randomly allocated into four groups of 20 chicks each. The control group was intraperitoneally inoculated with normal saline at 1 day old, and the other three infected groups were intraperitoneally injected with LN 1201 virus solution at 1 day old, with 3000 TCID_50_/chicken. The TF was administered orally for 3 consecutive days at 2–4 days old, with the low- and high-dose TF groups receiving 0.25 and 0.5 mL/chicken per day, respectively; the TF was not administered in the infection control group. All the chickens were weighed at 7, 14, 21, and 28 days old, and anticoagulated blood and cloacal cotton swabs were collected for detection of REV viral load. At 28 days old, all the chickens were dissected, and the development index of immune organs was measured. This scenario aligns with the need for emergency intervention after early infection of chicks in breeding sites, focusing on the therapeutic value of TF.

### 2.4. Suppressing the Effect of TF on REV Congenital Infection via Embryonic Yolk Sac Inoculation

**Figure 1:**
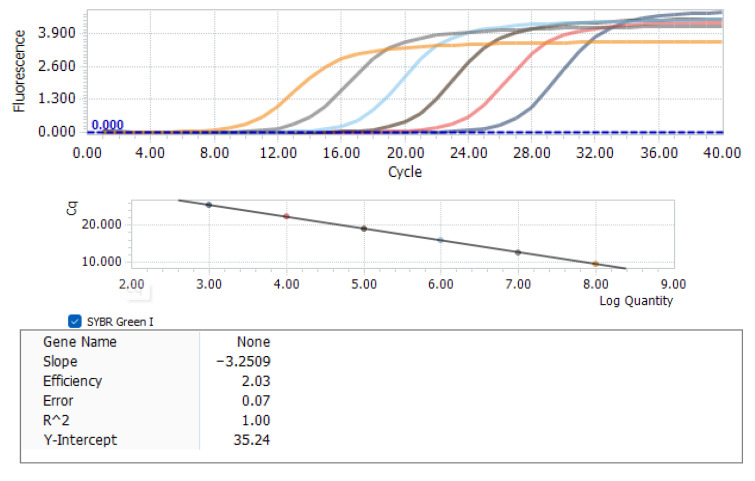
Standard curves and amplification curves.In the amplification curves (upper panel), colors represent serial 10-fold dilutions of the REV gag gene plasmid from left to right: orange (10^−2^ copies/μL), gray (10^−3^ copies/μL), light blue (10^−4^ copies/μL), brown (10^−5^ copies/μL), pink (10^−6^ copies/μL), dark blue (10^−7^ copies/μL).

A total of 120 SPF chicken embryos were inoculated with LN1201 virus solution at 100 TCID_50_/embryo via the yolk sac at 7 embryonic days old. This yolk sac inoculation method serves as a simulated vertical transmission model to mimic the congenital REV infection process in chicks. The SPF chicken embryos at 7 days of embryonic development were inoculated with sterile physiological saline as a blank control. The incubation parameters were as follows: incubation temperature 37.8 °C, relative humidity 55–65%, and egg-turning frequency once every 2 h. After hatching, the REV-positive chicks were screened according to the fluorescence quantitative PCR detection method established in our laboratory. In this study, absolute quantification was used for qPCR detection, with the REV gag gene as the target. The amplification primers were qREVpol-F (CCCATTCATGTCCAGCTAT) and qREVpol-R (AGGGAGGAGAGGAGTGTTCC). The recombinant plasmid containing the REV gag gene was serially diluted 10-fold across a gradient of 6 concentrations (10^2^–10^7^ copies/μL) to generate a standard curve, with an R^2^ value of 1. Standard curve parameters were as follows (Figure 1): slope = −3.2509, amplification efficiency = 103% (efficiency = (10^(−1/slope)−1) × 100%; 10^(−1/slope) ≈ 2.03), consistent with R^2^ = 1 and falling within the ideal qPCR efficiency range (90–110%), ensuring reliable REV quantification. The limit of detection (LOD) was 1 × 10^2^ copies/μL, and the limit of quantitation (LOQ) was 1 × 10^3^ copies/μL. During the extraction stage, a positive control (chicken liver tissue spiked with a known amount of REV) and a negative control (sterile PBS) were set up to eliminate errors and contamination. Then, the REV-positive chicks were randomly assigned into 3 groups. The control group was not treated with TF. The chickens in the single-dose group were treated with TF at 0.50 mL/chicken every day for 3 consecutive days at 2–4 days old. The chickens in the multiple-dose group were treated with TF at 0.50 mL/chicken every day for 3 straight days at the ages of 2–4 days and 7–9 days. All chickens were weighed at 7, 14, and 21 days old, and anticoagulated blood and cloacal swabs were collected at 5, 10, 15, and 20 days old to detect the REV viral load. At 21 days old, all the chickens were dissected, and the development index of immune organs was measured. This scenario addresses the challenge of congenital infections caused by the vertical transmission of REV and evaluates the intervention effect of TF on such high-risk chicks.

### 2.5. Detection of Various Pathogenic Indicators

The body weight data at the same time point are expressed as the mean ± standard deviation, and the comparison of differences between groups was performed using analysis of variance (ANOVA) to observe the inhibitory effect of the viral infection on the growth and TF’s mitigating effect on the growth inhibition caused by the viral infection. The liver, spleen, thymus, and bursa of Fabricius were collected and weighed at each slaughter and calculated according to the following formula: immune organ development index (%) = immune organ weight (g)/chick weight (g) × 100%. RNA was extracted from anticoagulated blood (100 μL) and cloacal swabs (eluted with 500 μL of saline) using an OMEGA RNA kit (It is produced by Omega Bio-Tek, a company located in Norcross, GA, USA). The reverse transcription steps were as follows: First, a 10 μL system (containing gDNA Clean Reaction Mix, viral RNA, and RNase-free water) was reacted at 42 °C for 2 min to remove residual genomic DNA in the RNA to avoid false positives. Then, 10 μL of the above reaction solution was taken, and reverse transcription buffer and RNase-free water were added to construct a 20 μL system. The reaction was carried out at 37 °C for 15 min to complete the reverse transcription of RNA into cDNA, followed by a reaction at 85 °C for 5 s to inactivate the reverse transcriptase. Finally, the cDNA product was stored at −20 °C for later use.

For REV-specific qPCR (lab-established), we used pol gene primers (F: 5′-CCCATTCATGTCCAGCTAT-3′, R: 5′-AGGGAGGAGAGGAGTGTTCC-3′) in a 20 µL system (10 µL of 2 × SYBR Mix, 0.8 µL of each primer, 2 µL of RNA, 6.4 µL of nuclease-free water) with the following conditions: 95 °C for 3 min and 40 cycles of 95 °C 10 s/60 °C 30 s. The positive rates (%) and viral load (copy/mL) were counted. Chicks/embryos were assigned to each group using the block randomization method, with the randomization scheme generated and sealed by independent statisticians. The personnel responsible for body weight measurement (using an electronic scale), autopsy (organ weighing), and qPCR testing (result interpretation) were all blinded (unaware of group allocation information), in compliance with the CONSORT guidelines for animal experiments.

### 2.6. Statistical Analysis

Experimental data were statistically analyzed using the GraphPad Prism 5 software. The Trend Log-rank test was used for statistical comparison of survival trends (e.g., chick mortality in different groups). For mortality data in the vertical transmission experiment, Cox’s proportional hazards regression was further applied to quantify the effect of TF on mortality risk, with “survival time of chicks (days)” and “event status (1 = death, 0 = survival)” as outcome variables, and “group” as the fixed factor. Survival time indices (median, mean, 95% CI) and hazard ratios (HRs) with 95% CI were reported to characterize the protective effect of TF. For two-group comparisons, T-tests (for normally distributed data) or nonparametric tests (for non-normally distributed data) were applied; for single-factor multi-group comparisons, one-way ANOVA (or nonparametric tests/mixed models if applicable) was adopted; for data involving two factors (e.g., the combined effects of “group” and “time” on longitudinal indices like chick body weight and REV viral load), two-way ANOVA (or mixed models to address within-subject correlation in repeated measurements) was used. All multiple comparisons between groups were performed using Tukey’s HSD test, and a corrected *p* ≤ 0.05 was defined as a significant difference (* *p* < 0.05, ** *p* < 0.01, *** *p* < 0.001, **** *p* < 0.0001). Statistical significance was determined as *p* ≤ 0.05.

## 3. Results

### 3.1. Preventive Effect of TF on REV Infection

**Figure 2:**
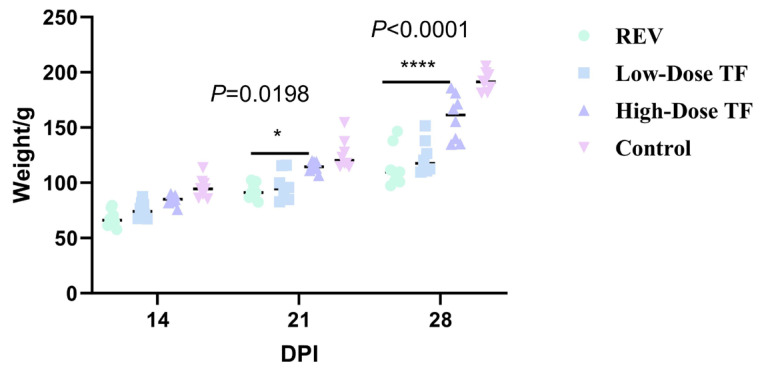
Comparison of body weight at different ages in different groups. Groups: control (blank control group, no reticuloendotheliosis virus (REV) infection or transfer factor (TF) treatment); REV (REV infection control group, infected with REV but no TF treatment); low-dose TF (REV-infected group treated with low-dose TF); high-dose TF (REV-infected group treated with high-dose TF). Different symbols in the column diagrams indicate statistically significant differences (all multiple comparisons between groups were performed using Tukey’s HSD test, and a corrected *p* ≤ 0.05 was defined as a significant difference; * *p <* 0.05, **** *p <* 0.0001).

**Figure 3:**
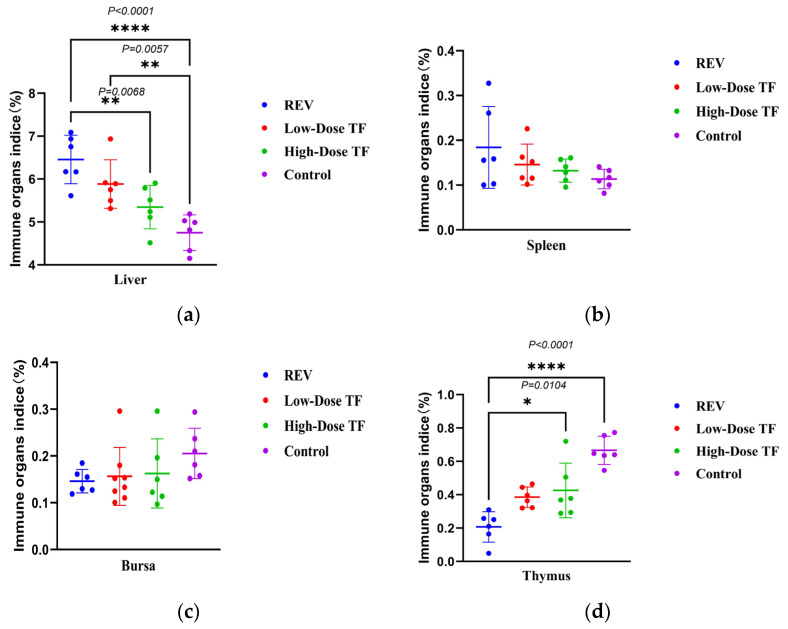
Comparison of organ development indices at 28 days in different groups: (**a**) liver; (**b**) spleen; (**c**) bursa; (**d**) thymus. Different symbols in the column diagrams indicate statistically significant differences (all multiple comparisons between groups were performed using Tukey’s HSD test, and a corrected *p* ≤ 0.05 was defined as a significant difference; * *p* < 0.05, ** *p* < 0.01, **** *p* < 0.0001).

The pre-administration of TF significantly relieved the inhibitory effect of REV infection on body weight gain. Figure 2 shows that the average body weight of the blank control group at 28 days old was 191.1 ± 8.7 g, while that of the REV infection control group without TF was 114 ± 18 g. The difference between the two groups was highly significant. Meanwhile, the body weight of the high-dose TF group was significantly higher than that of the REV-infected control group (*p <* 0.0001), indicating that TF could alleviate the inhibition of body weight gain caused by REV infection. At the same time, TF significantly alleviated the organ development changes caused by REV infection. Figure 3 shows that the liver development index of the REV infection control group (6.45 ± 0.56%) was significantly increased compared with the blank group (4.74 ± 0.41%) (*p* ≤ 0.0001), and the thymus development index (0.2 ± 0.09%) was significantly decreased compared with the blank group (0.42 ± 0.16%) (*p* ≤ 0.0001), indicating that REV infection caused liver enlargement and thymus atrophy in the chickens. However, compared with the REV-infected control group, the liver and thymus development indices of the high-dose TF group were significantly improved (*p <* 0.01). Notably, the thymus development index of the high-dose TF group (0.69 ± 0.06%) was not significantly different from that of the control group (0.42 ± 0.16%), while the liver development index (5.34 ± 0.50%), although still differing from the control group (4.74 ± 0.41%), showed a substantial trend toward restoration, collectively indicating that TF alleviates REV-induced liver enlargement and thymus atrophy (Supplementary Materials, File S1).

**Figure 4:**
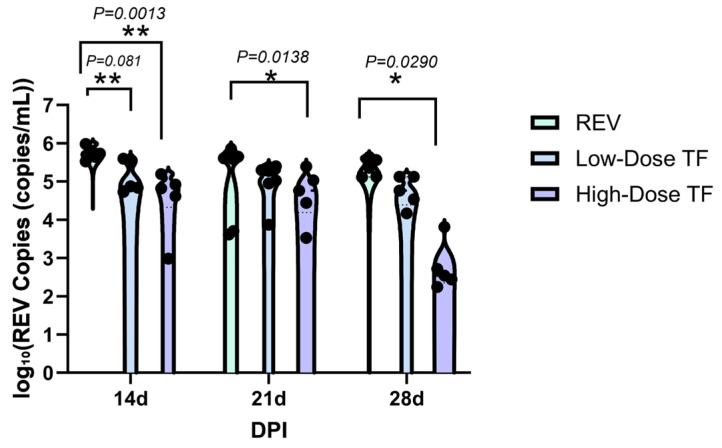
Comparison of REV copy number in the blood of chickens at different time points. Black dots represent individual sample data points. Different symbols in the column diagrams indicate statistically significant differences (all multiple comparisons between groups were performed using Tukey’s HSD test, and a corrected *p* ≤ 0.05 was defined as a significant difference; * *p* < 0.05, ** *p* < 0.01).

**Figure 5:**
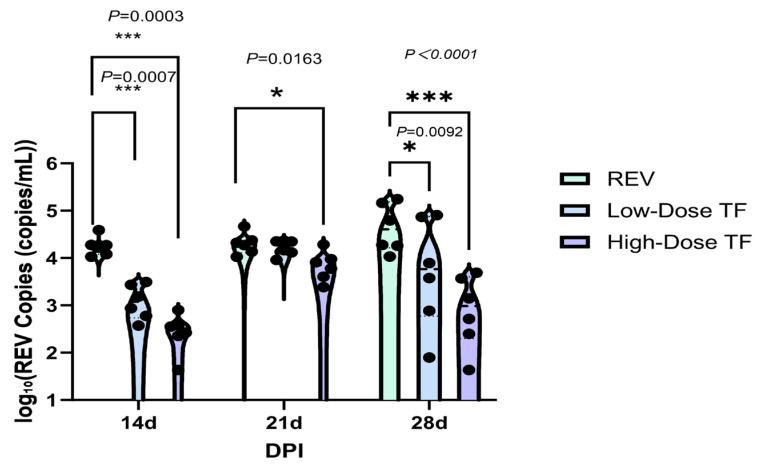
Comparison of REV copy number in the cloaca of chickens at different time points. Black dots represent individual sample data points. Different symbols in the column diagrams indicate statistically significant differences (all multiple comparisons between groups were performed using Tukey’s HSD test, and a corrected *p* ≤ 0.05 was defined as a significant difference; * *p* < 0.05, *** *p* < 0.001).

Table 1 shows that REV nucleic acid was not detected in the blood and cloacal cotton swabs of the blank control group throughout the whole experimental period, indicating that there was no infection. In the REV infection control group, REV positivity was consistently maintained. For example, at 28 days old, the positive rates of REV nucleic acid in the blood and cloacal swabs were 100%, while in the high-dose TF group at 28 days old, the positive rates of REV nucleic acid in the blood and cloacal swabs were 50% and 57%, respectively. In addition, even in similarly infected chickens, the TF prophylaxis significantly reduced the REV viral load relative to the REV-infected controls specifically in the blood at 14d (Figure 4) and in the cloaca at 14d and 28d (Figure 5), confirming that TF at both doses inhibited REV replication in peripheral blood and cloacal virus shedding in SPF chicks. The high-dose transfer factor had a better effect.

### 3.2. Inhibitory Effect of TF on REV Horizontal Transmission

**Figure 6:**
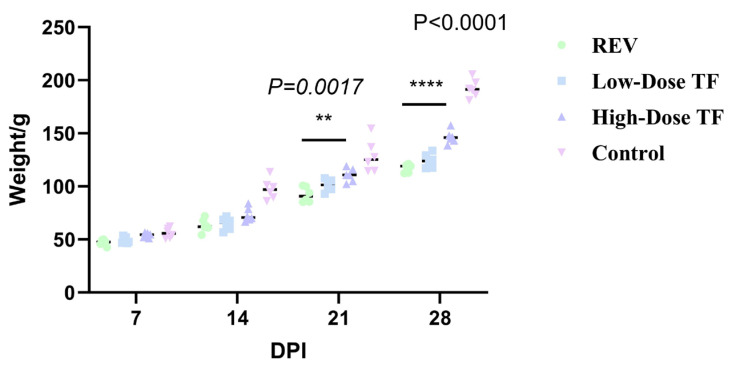
Comparison of body weight at different ages in different groups. Different symbols in the column diagrams indicate statistically significant differences (all multiple comparisons between groups were performed using Tukey’s HSD test, and a corrected *p* ≤ 0.05 was defined as a significant difference; ** *p* < 0.01, **** *p* < 0.0001).

**Figure 7:**
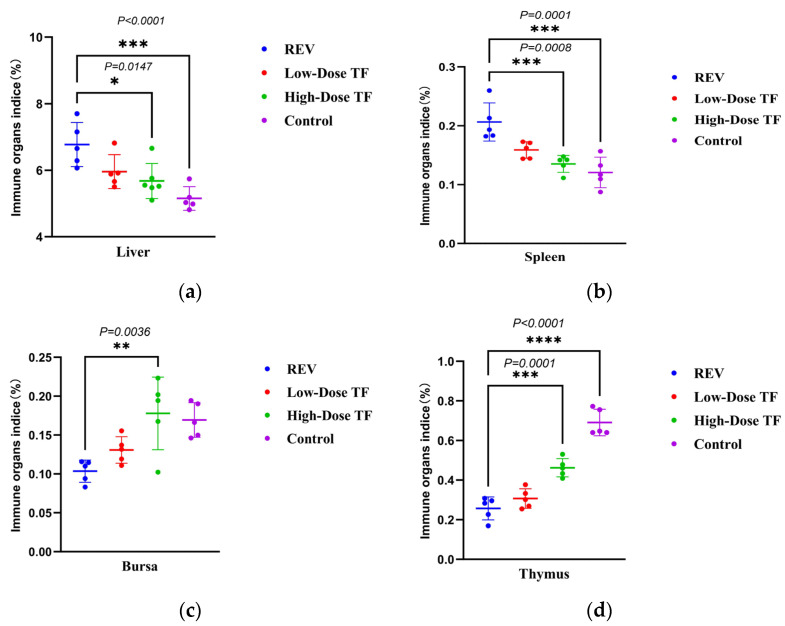
Comparison of organ development indices at 28 days in different groups: (**a**) liver; (**b**) spleen; (**c**) bursa; (**d**) thymus. Different symbols in the column diagrams indicate statistically significant differences (all multiple comparisons between groups were performed using Tukey’s HSD test, and a corrected *p* ≤ 0.05 was defined as a significant difference; * *p* < 0.05, ** *p* < 0.01, *** *p* < 0.001, **** *p* < 0.0001).

TF significantly relieved the inhibition of REV infection on body weight gain after 1 day of REV infection. Figure 6 shows that the body weight of the REV-infected control group was consistently lower than that of the blank control group at 21 and 28 days. The average body weight of the blank control group was 192.2 ± 8.5 g at 28 days old, while that of the REV-infected control group was only 117 ± 3.8 g. However, the body weight of the high-dose TF group was significantly higher than that of the REV-infected control group (*p* < 0.0001), indicating that TF could alleviate the inhibition of body weight gain caused by REV infection. The organ development index also showed that TF significantly alleviated the effect of REV infection on organ development. Figure 7 shows that the liver (6.77 ± 0.66%) and spleen (0.2 ± 0.03%) development index of the REV infection control group were significantly increased compared with the blank group (5.15 ± 0.35%, 0.12 ± 0.02%), and the thymus development index was significantly decreased compared with the blank group (*p* < 0.0001), indicating that REV infection caused liver and spleen enlargement and thymus atrophy in the chickens. However, there was no significant difference in the development index of three organs between the high-dose TF and control groups. Still, there was a substantial difference between the high-dose TF (0.46 ± 0.04%) and control groups (0.69 ± 0.06%) (*p* < 0.01), indicating that TF alleviated the hepatosplenomegaly and thymus atrophy of the SPF chicks caused by REV infection. At the same time, the bursa development index of the high-dose TF group (0.17 ± 0.04%) was significantly higher than that of the REV-infected control group (0.10 ± 0.01%) (*p* < 0.01), indicating that the TF alleviated the bursa atrophy caused by REV infection.

**Figure 8:**
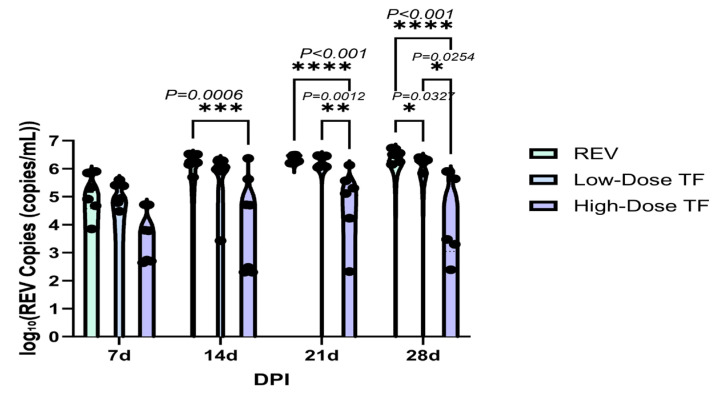
Comparison of REV copy number in the blood of chickens at different time points. Black dots represent individual sample data points. Different symbols in the column diagrams indicate statistically significant differences (all multiple comparisons between groups were performed using Tukey’s HSD test, and a corrected *p* ≤ 0.05 was defined as a significant difference; * *p* < 0.05, ** *p* < 0.01, *** *p* < 0.001, **** *p* < 0.0001).

**Figure 9:**
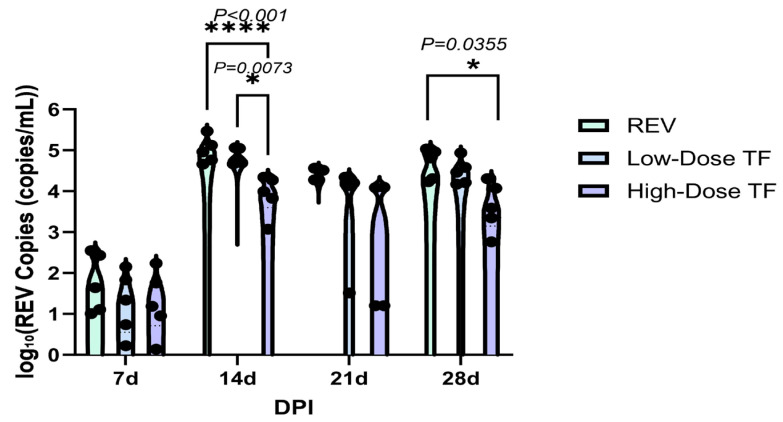
Comparison of REV copy number in the cloaca of chickens at different time points. Black dots represent individual sample data points. Different symbols in the column diagrams indicate statistically significant differences (all multiple comparisons between groups were performed using Tukey’s HSD test, and a corrected *p* ≤ 0.05 was defined as a significant difference; * *p* < 0.05, **** *p* < 0.0001).

Table 2 shows that REV nucleic acid was not detected in the blood and cloaca swabs of the blank control group throughout the experimental period, indicating that there was no REV infection, and REV positivity was maintained in the blood of the REV-infected control group. At the early stage of infection, such as at 7 days old, the positive rate of REV nucleic acid in the blood and cloaca swabs of the TF group was lower than that of the REV infection control group and was positively correlated with the TF dose. Even in similarly infected chickens, those treated with TF showed a significant reduction in the REV viral load compared with the REV-infected control group in the blood (Figure 8) and cloaca (Figure 9), demonstrating that TF at both doses inhibited REV replication in peripheral blood and cloacal virus shedding in the SPF chicks. For example, the REV viral load in peripheral lymphocytes of the high-dose TF group was significantly lower than that of the REV infection at 14, 21, and 28 d (*p <* 0.05), and the difference was highly significant at 21 and 28 d (*p <* 0.001). At 14 days, the virus load of the cloaca swab in the high-dose TF group was lower than that in the REV group (*p <* 0.001).

### 3.3. Inhibitory Effect of TF on REV Congenital Infection via Embryonic Yolk Sac Inoculation

**Figure 10:**
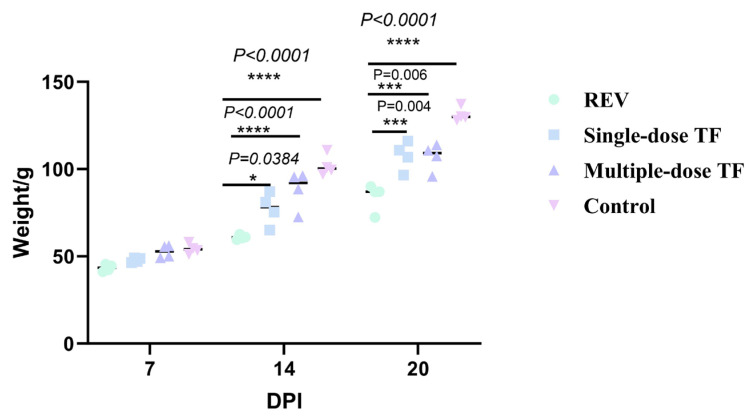
Comparison of body weight at different ages in different groups. Groups: control (blank control group, no reticuloendotheliosis virus (REV) infection or transfer factor (TF) treatment); REV (REV trans-egg infection control group, infected with REV via egg transmission but no TF treatment); single-dose TF (REV trans-egg infection group treated with single-course TF: 0.50 mL/chicken per day for 3 consecutive days at 2–4 days old); multiple-dose TF (REV trans-egg infection group treated with multiple-course TF: 0.50 mL/chicken per day for 3 consecutive days at both 2–4 days old and 7–9 days old). Different symbols in the column diagrams indicate statistically significant differences (all multiple comparisons between groups were performed using Tukey’s HSD test, and a corrected *p* ≤ 0.05 was defined as a significant difference; * *p* < 0.05, *** *p* < 0.001, **** *p* < 0.0001).

**Figure 11:**
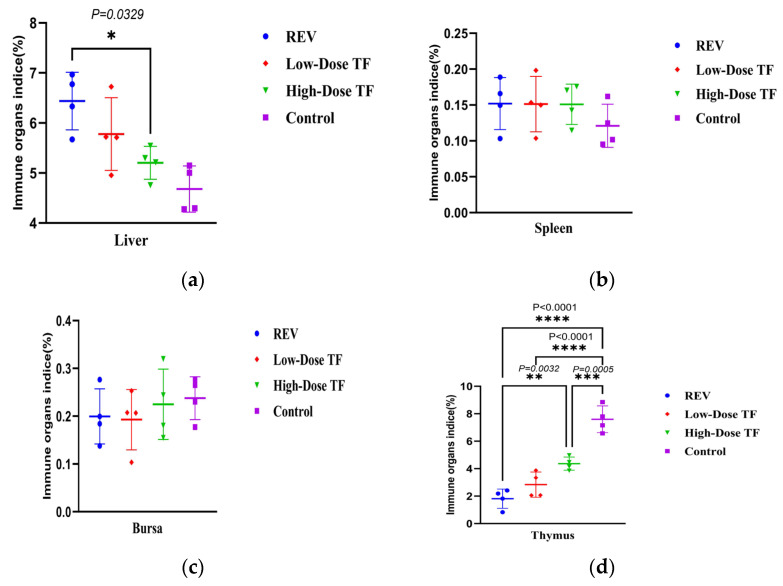
Comparison of organ development indices at 28 days in different groups: (**a**) liver; (**b**) spleen; (**c**) bursa; (**d**) thymus. Different symbols in the column diagrams indicate statistically significant differences (all multiple comparisons between groups were performed using Tukey’s HSD test, and a corrected *p* ≤ 0.05 was defined as a significant difference; * *p* < 0.05, ** *p* < 0.01, *** *p* < 0.001, **** *p* < 0.0001).

This experiment adopted a simulated vertical transmission model (via yolk sac inoculation at 7 embryonic days old) to evaluate TF’s inhibitory effect on REV infection transmitted from embryos to chicks. The reticuloendotheliosis virus (REV) has a significant inhibitory effect on chick growth after transmission through eggs: Figure 10 shows that, at 14 and 20 days old, the body weight of the REV-infected control group (60.95 ± 1.32 g, 84.07 ± 7.07 g) was extremely significantly lower than that of the blank control group (102.1 ± 6.01 g, 131.2 ± 4.04 g); at 14 days old, the body weight of the multiple-dose group (88.2 ± 10.96 g) was significantly higher than that of the REV-infected control group (60.95 ± 1.32 g) (*p* < 0.05); and at 20 days old, it (108.9 ± 4.27 g) was even extremely significantly higher than that of the REV-infected control group (84.0 ± 7.97 g) (*p* < 0.01), indicating that the use of TF significantly alleviated the inhibitory effect of REV infection via egg transmission on chick body weight gain. REV vertical transmission through eggs had a substantial impact on the development of the thymus in chicks: Figure 11 shows that, at 20 days old, the thymus development index of the REV-infected control group (1.81 ± 0.69 g) was extremely significantly lower than that of the blank control group (7.57 ± 0.97 g), and after timely intervention with TF, the thymus development index (2.83 ± 0.91 g, 4.36 ± 0.48 g) of the chicks was still significantly lower than that of the blank control group (7.57 ± 0.97 g) but significantly higher than that of the REV-infected control group (1.81 ± 0.69 g) without TF use, indicating that TF alleviated REV-induced thymic atrophy in the SPF chicks. Similarly, the liver development index of the chicks in the multiple-dose group (5.02 ± 0.32 g) was significantly lower than that of the REV-infected control group (6.43 ± 0.57 g) (*p <* 0.05), indicating that TF alleviated REV-induced hepatomegaly in the SPF chicks.

The vertical transmission of REV via eggs led to high mortality in chicks. At 20 days of age, the mortality rate of the REV-infected control group was 50.0%. For the TF-treated groups, the single-dose TF group had a mortality rate of 33.3%, representing a 16.7 % reduction and a 33.3% relative decrease compared to the REV-infected control group; the multiple-dose TF group had a mortality rate of 16.7%, corresponding to a 33.3 % reduction and a 66.6% relative decrease compared to the REV-infected control group.

**Figure 12:**
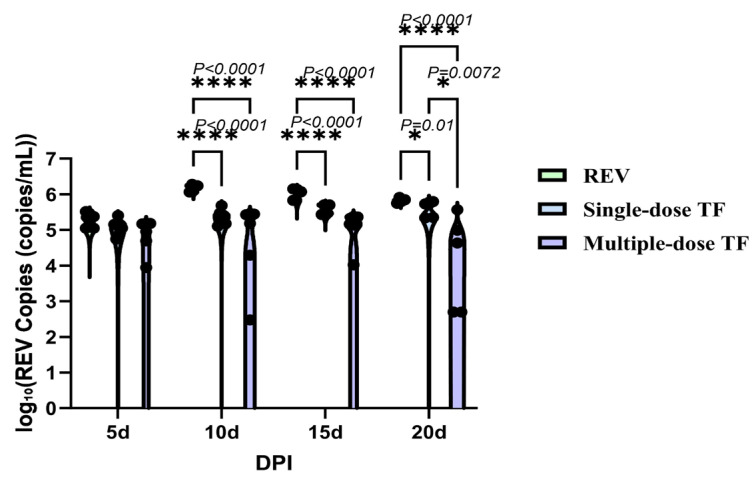
Comparison of REV copy number in the blood of chickens at different time points. Black dots represent individual sample data points. Different symbols in the column diagrams indicate statistically significant differences (all multiple comparisons between groups were performed using Tukey’s HSD test, and a corrected *p* ≤ 0.05 was defined as a significant difference; * *p* < 0.05, **** *p* < 0.0001).

**Figure 13:**
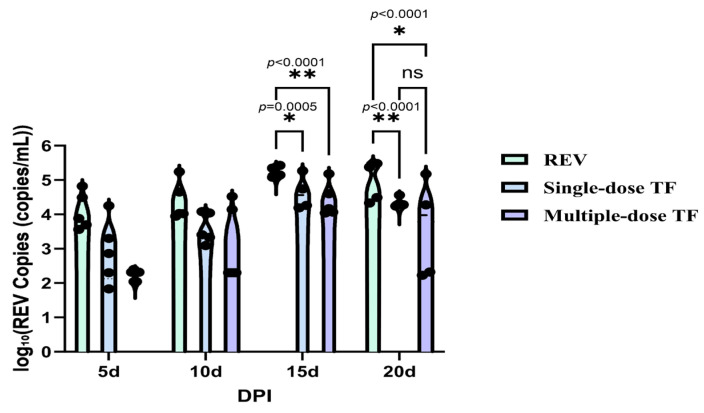
Comparison of REV copy number in the cloaca of chickens at different time points. Black dots represent individual sample data points. Different symbols in the column diagrams indicate statistically significant differences (all multiple comparisons between groups were performed using Tukey’s HSD test, and a corrected *p* ≤ 0.05 was defined as a significant difference; * *p* < 0.05, ** *p* < 0.01).

**Figure 14:**
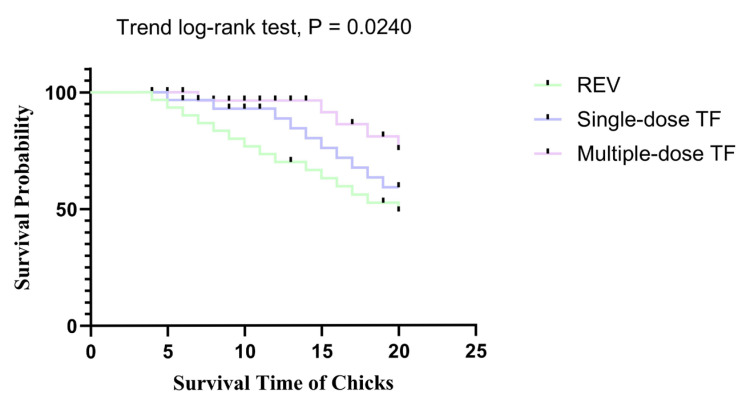
The survival curve of chicks in different treatment groups within 20 days of age (the Trend Log-rank test was used for statistical comparison, with a chi-square value of 5.098, df = 1, and *p =* 0.0240, indicating a significant trend in survival rates between the two groups).

Concurrently, the REV-positive rate (Table 3) in blood and cloacal swabs of the multiple-dose TF group was 60% at 20 days of age, which was consistent with the reduced mortality, further verifying that TF-mediated viral inhibition contributes to improved chick survival. Meanwhile, the use of TF also significantly inhibited REV replication in the blood and cloacal virus shedding. Figure 12 shows that at 10, 15, and 20 days, the number of REV nucleic acid copies in the blood of the REV-infected chicks in the multiple-dose TF group was significantly lower than that of the REV-infected control group without TF use; at the same time, the number of nucleic acid copies in the cloacal swabs of the REV-infected chicks in the multiple-dose TF group was also significantly lower than that of the REV-infected control group without TF use at 15 and 20 days (Figure 13). Statistical analysis via the Trend Log-rank test confirmed that the mortality reduction in the multiple-dose TF group was statistically significant (χ^2^ = 5.098, df = 1, *p =* 0.0240), with a clear dose-dependent trend, showing that multiple-dose TF was more effective than single-dose TF (Figure 14). Cox’s proportional hazards regression further quantified this effect: the REV control group had the shortest median survival time (12.0 days), while the multiple-dose TF group had the longest (18.0 days) (Table 4). Taking the REV control group as reference, the single-dose TF group had a significantly lower mortality risk (HR = 3.00, *p* = 0.033), and the multiple-dose TF group showed a similar trend (HR = 2.00) (Table 5). Thus, the single and multiple TF treatments showed significant inhibitory effects, and the inhibitory effect was significantly correlated with the number of uses.

## 4. Discussion

Avian reticuloendotheliosis virus and avian leukemia virus are immunosuppressive viruses that can be transmitted vertically through chick embryos. Both viruses can cause tumors in chickens [14,15,16,17]. In recent years, China has carried out a systematic eradication program against avian leukemia [18,19], and there have been few reports of avian leukemia virus detection. However, there have been no eradication measures against REV. Although there are many vaccine studies against REV, there is no commercial avian reticuloendotheliosis vaccine available worldwide for the prevention and control of the disease. To reduce the loss of chickens to REV, more measures are being explored for the prevention or treatment of the disease. For example, interferon has previously been shown to be effective at inhibiting replication of REV in vivo and in vitro [20].

In addition to interferon, transfer factor (TF) is also a bioactive substance related to immune regulation, usually a small-molecule mixture (polypeptide, nucleotide, etc.) extracted from sensitized animal lymphocytes. Transfer factors can transfer the immune memory of donors (such as the recognition ability of specific pathogens) to recipients, activate T lymphocytes, and enhance the response to particular antigens (such as viruses and tumors) [21,22]. Compared with interferon, TF has fewer side effects in clinical use, and the immune memory may be more enduring. Transfer factors have been widely used in the prevention and treatment of poultry diseases and have been clinically proven to have promising therapeutic effects in the treatment of infectious bursal disease, avian influenza, and Marek’s disease. Therefore, the feasibility of TF controlling REV was investigated in this study, especially the inhibition of REV infection in different ways.

Post-infection weakness and stunted growth in chicks are typical pathogenic characteristics of REV. Previous studies have demonstrated that TFs can effectively promote weight gain in broilers, enhance nutrient absorption by increasing the villus height and crypt depth, and regulate intestinal microbiota dysbiosis [9]. This study demonstrated that, regardless of the route of REV infection, it leads to growth inhibition in chickens, which is significantly different from that in uninfected chickens. In the parenteral infection models described in Section 2.2 and Section 2.3 of this study, the dose of 3000 TCID_50_ per chick for REV-LN1201 was selected based on two aspects. On the one hand, it refers to the clinical detection data obtained by Xu et al. (2023) [20], which is consistent with the plasma REV load range in naturally infected broilers and aligns with field reality. On the other hand, pre-experimental verification showed that this dose can induce typical REV pathogenic effects such as growth inhibition and thymic atrophy, with an acute mortality rate of <20%, which not only ensures the stability of the infection model but also avoids the interference of high mortality on the evaluation of TF’s intervention effect. TF treatment can effectively alleviate REV-induced growth inhibition. These findings indicate that TF has application potential in reducing clinical economic losses caused by REV infection. In addition to the inhibitory effect on production performance, the inhibitory effect on immune function is a more critical aspect of REV harm, and there have been many reports focusing on the immunosuppression caused by REV infection [11,23,24]. This study further confirmed that REV caused thymus atrophy and liver damage after three routes of infection, while TF could effectively alleviate the thymus atrophy and liver damage caused by REV infection. The thymic gland is a key organ in the immune system, and it is the core site of T lymphocyte differentiation and maturation. Thymosin, thymopoietin, and other hormones secreted by thymic epithelial cells promote T cell proliferation, differentiation, and functional maturity [25,26,27]. The thymus of chicks develops rapidly after hatching and is the core of early immune system establishment. Thymic hypoplasia can lead to severe immune deficiency [12]. A TF can effectively alleviate thymus atrophy caused by REV infection, which helps to enhance immunity at a young age and improve resistance to the virus in the early stage of viral infection.

While interferon exerts direct antiviral and antitumor effects by regulating immune responses, TF primarily mediates specific immune enhancement by transmitting immune sensitization information; our laboratory has previously confirmed that TF in SPF chickens can promote the secretion of multiple cytokines and enhance cellular immune function, thereby inhibiting viral replication [28]. The results of this study confirmed that REV replication in vivo was significantly inhibited regardless of whether TF was used prior to or after REV infection. Among various infection routes, the harm caused by vertical transmission of REV through eggs was undoubtedly the greatest. In addition to causing early damage to immune organs, REV vertical transmission via eggs can also lead to persistent viremia. For example, 100% of the chicks in all groups showed REV-positive blood at 7 days old after chicken embryo inoculation. Although neither low- nor high-dose TF could completely suppress the early infection peak induced by REV vertical transmission, they significantly reduced mortality and inhibited cloacal viral shedding. In actual production, the REV vertical transmission probability through eggs is not high, but young chicks are susceptible to REV, and they will spread horizontally to more chickens through the cloaca. TFs can inhibit cloacal virus shedding, which helps reduce the probability of REV horizontal transmission during brooding. This study is the first to confirm the inhibitory effect of TF on REV, and its effect is consistent with the performance of TF in other avian viruses. Guo et al. [8] found that porcine-derived TF can reduce the load of chicken infectious anemia virus by 30–40%, which is similar to the trend of TF reducing REV load by 50–68% in this study. However, this study has two innovations: first, it covers three scenarios of “prevention–post-infection treatment–congenital infection”, which is more comprehensive than previous studies with a single treatment scenario; second, it confirms that TF can inhibit cloacal virus shedding, which has not been reported in studies on TF against avian influenza virus, suggesting that TF may block the horizontal transmission of REV by reducing detoxification.

This study has limitations: The vertical transmission of REV could not be completely blocked, and the inhibition of viral replication was incomplete. In our future research, we will focus on three directions: first, dose optimization will be carried out, which involves exploring the safety and long-term efficacy of high-dose transfer factor (TF) at above 0.5 mL; second, a combination strategy will be used, specifically testing the synergistic effect between TF and low-dose interferon to determine whether it can further enhance the reticuloendotheliosis virus (REV) inhibition rate; and third, mechanism analysis will be conducted, that is, screening key pathways regulated by TF (such as the TLR-3/IFN-β pathway) via RNA sequencing (RNA-seq) and clarifying the molecular mechanism by which TF regulates T cell subsets.

In summary, this study verified that TF played a role in alleviating growth inhibition and organ development damage caused by REV infection through different infection routes. It significantly reduced the mortality rate and could effectively inhibit viral replication in vivo and cloacal virus shedding. In particular, its use in chicks at a low age may play a more important role. Before effective measures for REV eradication are established, this study only confirms that TF can effectively inhibit REV replication in chicks under laboratory artificial infection models; it does not claim to solve natural REV vertical or horizontal transmission problems. The results suggest that TF may serve as a potential auxiliary measure for reducing REV propagation in poultry production, but its actual application effect needs to be further verified by field trials.

## Figures and Tables

**Table 1:** REV positivity rate in plasma and cloaca of different groups in a preventive experiment.

Day of Age	REV-Infected Control Group		Low-Dose TF Group		High-Dose TF Group		Blank Control Group	
	Plasma	Cloacal Cavity	Plasma	Cloacal Cavity	Plasma	Cloacal Cavity	Plasma	Cloacal Cavity
14 d	8/8 (100%)	6/8 (75%)	8/8 (100%)	6/8 (75%)	8/8 (100%)	4/8 (50%)	0/8 (0%)	0/8 (0%)
21 d	7/7 (100%)	7/7 (100%)	5/6 (83%)	7/7 (100%)	7/7 (100%)	6/7 (85%)	0/8 (0%)	0/8 (0%)
28 d	6/6 (100%)	7/7 (100%)	6/6 (100%)	6/7 (85%)	3/6 (50%)	4/7 (57%)	0/8 (0%)	0/8 (0%)

Groups: blank control group (no reticuloendotheliosis virus (REV) infection or transfer factor (TF) treatment); REV-infected control group (infected with REV but no TF treatment); low-dose TF group (REV-infected group treated with low-dose TF: 0.25 mL/chicken per day for 3 consecutive days at 2–4 days old); high-dose TF group (REV-infected group treated with high-dose TF: 0.5 mL/chicken per day for 3 consecutive days at 2–4 days old). Data are presented as “number of positive chicks/number of tested chicks (positive rate)”, where “plasma” and “cloacal cavity” represent the detection samples for REV positivity, and “day of age” indicates the sampling time points (14 d, 21 d, 28 d).

**Table 2:** REV positivity in plasma and cloaca of different groups in horizontal transmission experiments.

Day of Age	REV-Infected Control Group		Low-Dose TF Group		High-Dose TF Group		Blank Control Group	
	Plasma	Cloacal Cavity	Plasma	Cloacal Cavity	Plasma	Cloacal Cavity	Plasma	Cloacal Cavity
7 d	8/8 (100%)	2/8 (25%)	7/8 (88%)	0/8 (0%)	6/8 (75%)	0/8 (0%)	0/6 (0%)	0/8 (0%)
14 d	8/8 (100%)	8/8 (100%)	7/8 (88%)	8/8 (100%)	4/8 (50%)	8/8 (100%)	0/6 (0%)	0/8 (0%)
21 d	8/8 (100%)	8/8 (100%)	8/8 (100%)	7/8 (88%)	6/8 (75%)	7/8 (88%)	0/6 (0%)	0/8 (0%)
28 d	8/8 (100%)	8/8 (100%)	8/8 (100%)	8/8 (100%)	7/8 (88%)	6/8 (75%)	0/6 (0%)	0/8 (0%)

**Table 3:** Prevalence of REV positivity in plasma and cloaca of different groups in transovarian transmission assay.

Dayof Age	REV-Infected Control Group		Single-Dose TF Group		Multiple-Dose TF Group		Blank Control Group	
	Plasma	Cloacal Cavity	Plasma	Cloacal Cavity	Plasma	Cloacal Cavity	Plasma	Cloacal Cavity
5 d	6/6 (100%)	5/6 (83%)	6/6 (100%)	3/6 (50%)	6/6 (100%)	2/6 (33%)	0/6 (0%)	0/6 (0%)
10 d	4/4 (100%)	4/4 (100%)	6/6 (100%)	4/6 (100%)	5/6 (83%)	2/6 (33%)	0/6 (0%)	0/6 (0%)
15 d	3/3 (100%)	3/3 (100%)	4/4 (100%)	4/4 (100%)	5/5 (100%)	5/5 (100%)	0/6 (0%)	0/6 (0%)
20 d	3/3 (100%)	3/3 (100%)	4/4 (100%)	4/4 (100%)	3/5 (60%)	3/5 (60%)	0/6 (0%)	0/6 (0%)

**Table 4:** Survival time indices of chicks in REV vertical transmission experiment via embryonic yolk sac inoculation.

Group	Median Survival Time (Days)	95% CI	Average Survival Period (Days)	95% CI
REV	12	6.9–17.1	12.3	9.8–14.9
Single-dose TF	15	11.9–18.1	14.7	12.5–16.9
Multiple-dose TF	18	14.0–22.0	17.3	14.9–19.6

**Table 5:** Cox’s proportional hazards regression results for chick mortality in REV vertical transmission experiment.

Group	B	SE	Wald	df	Sig.	Exp(B)
REV			4.640	2	0.098	
Single-dose TF	1.099	0.516	4.526	1	0.033	3.000
Multiple-dose TF	0.693	0.548	1.602	1	0.206	2.000

## Data Availability

Data is available on request from corresponding authors. The raw data are not publicly available due to laboratory’s policies and confidential agreements.

## Supplementary Materials

The following supporting information can be downloaded at: https://www.mdpi.com/article/10.3390/vetsci12111041/s1, File S1.

## Abbreviations

The following abbreviations are used in this manuscript:

REV	Reticuloendotheliosis Virus
TF	Transfer Factor
RE	Reticuloendotheliosis
SPF	Specific Pathogen-Free
TCID_50_	Tissue Culture Infectious Dose 50
PCR	Polymerase Chain Reaction
LOD	Limit of Detection
LOQ	Limit of Quantitation
