# Supplementary figures and images for: Inhibitory Effect of Transfer Factor on Avian Reticuloendotheliosis Virus Infection in Chicks
Source: Vet Sci. 2025 Oct 31;12(11):1041. doi: 10.3390/vetsci12111041 (PMC12656777; doi:10.3390/vetsci12111041)

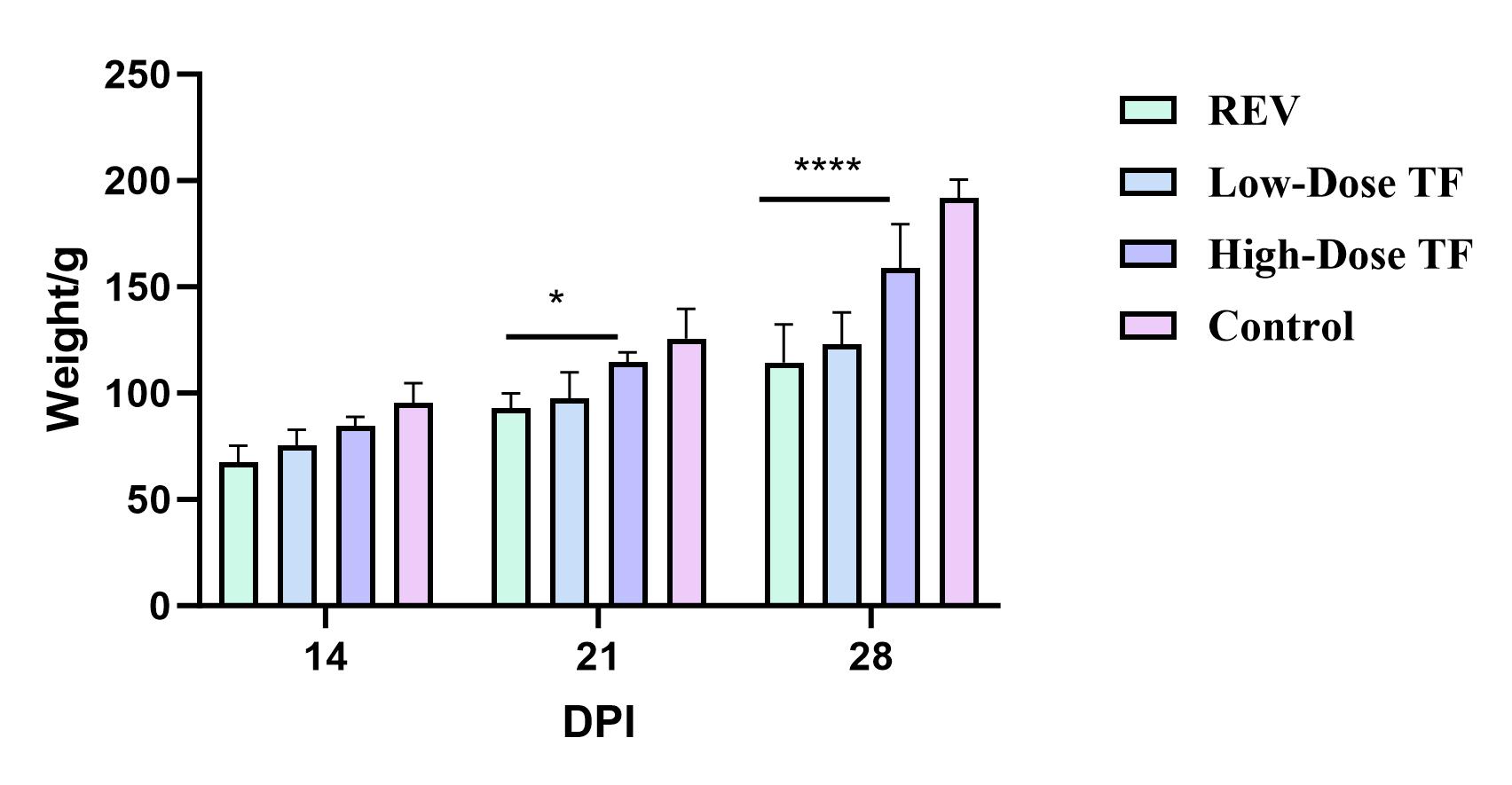

Supplement: Supplementary file 1 [file vetsci-12-01041-s001.zip › figure S1.jpg]

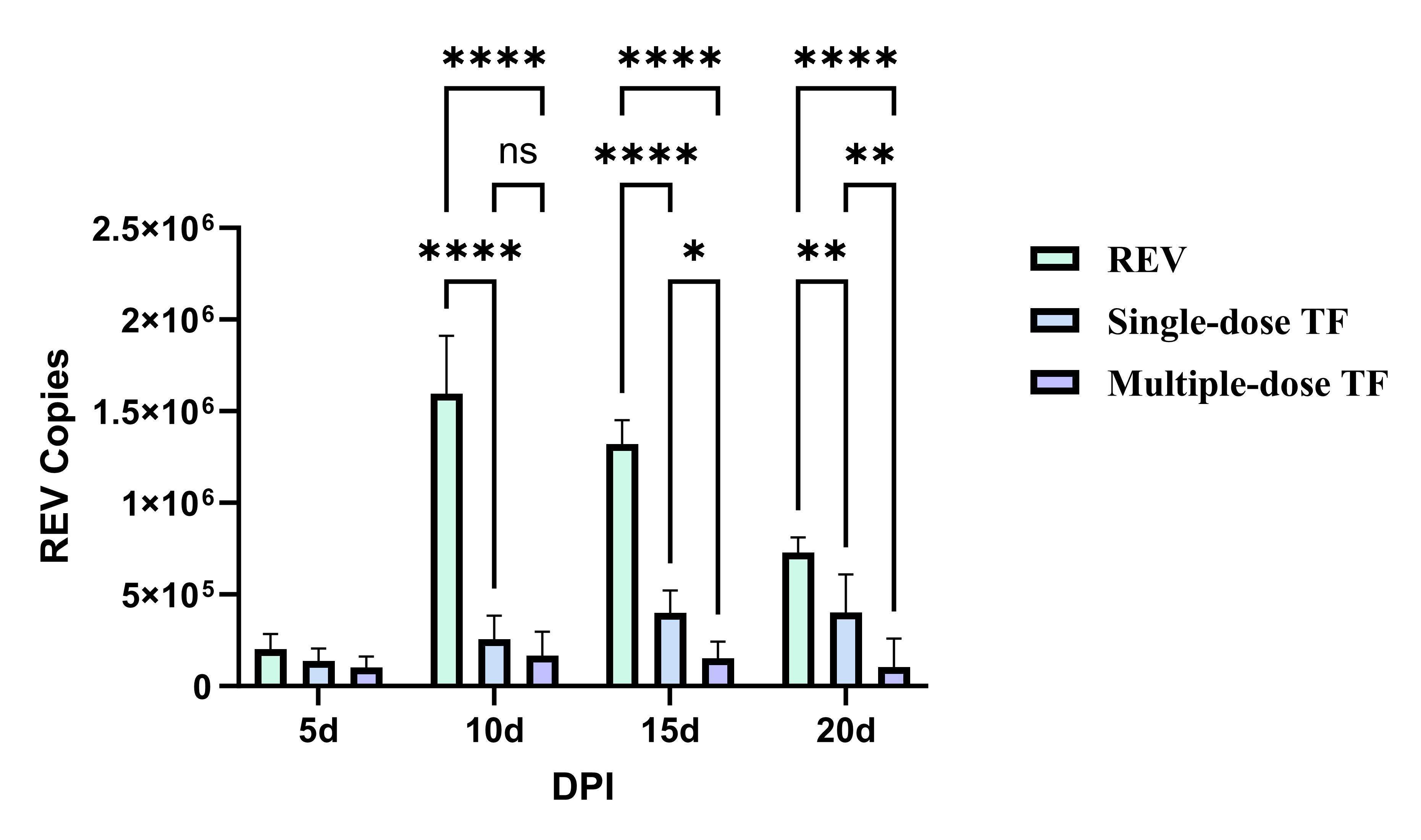

Supplement: Supplementary file 1 [file vetsci-12-01041-s001.zip › figure S2.jpg]

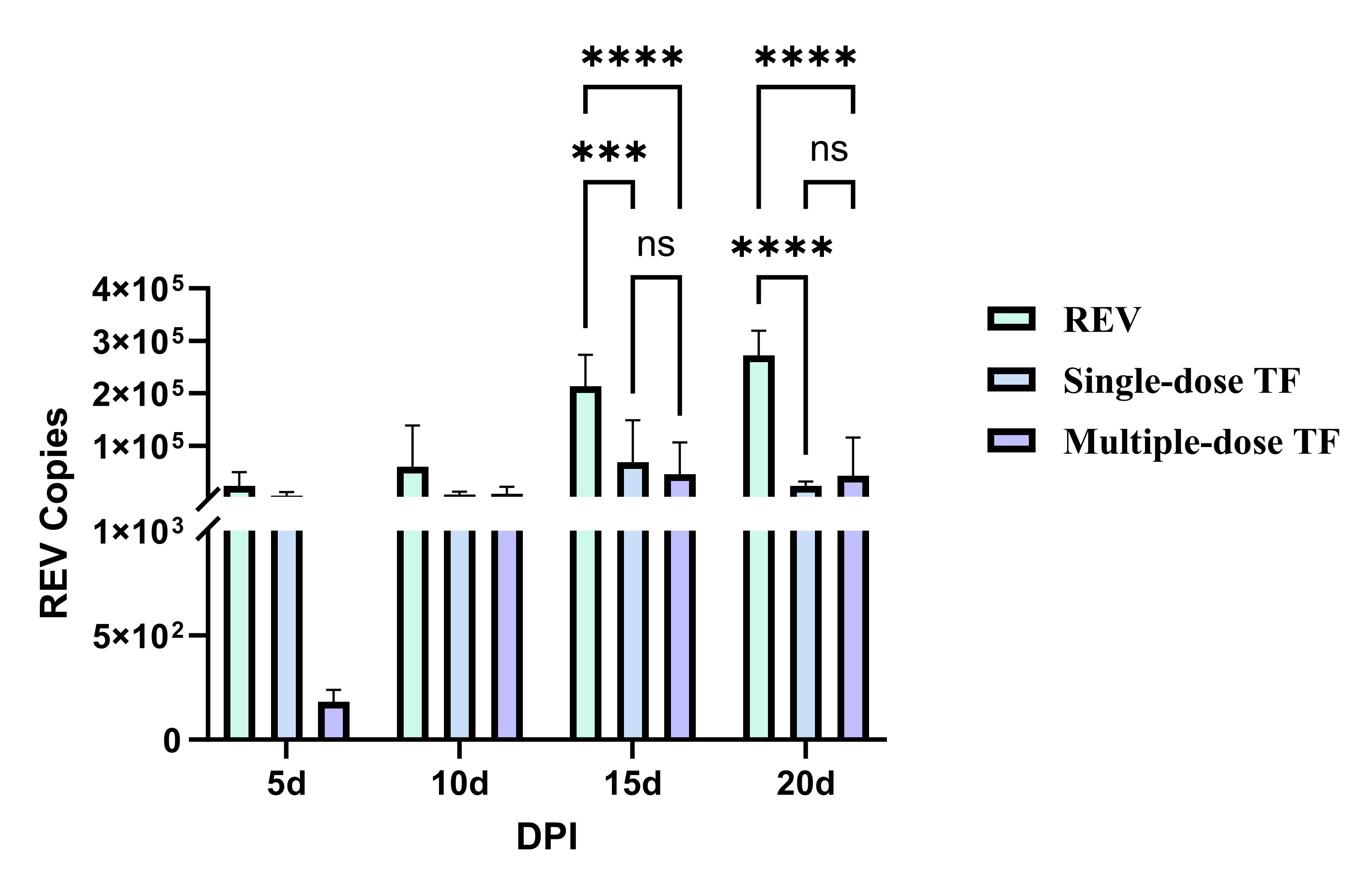

Supplement: Supplementary file 1 [file vetsci-12-01041-s001.zip › figure S3.jpg]

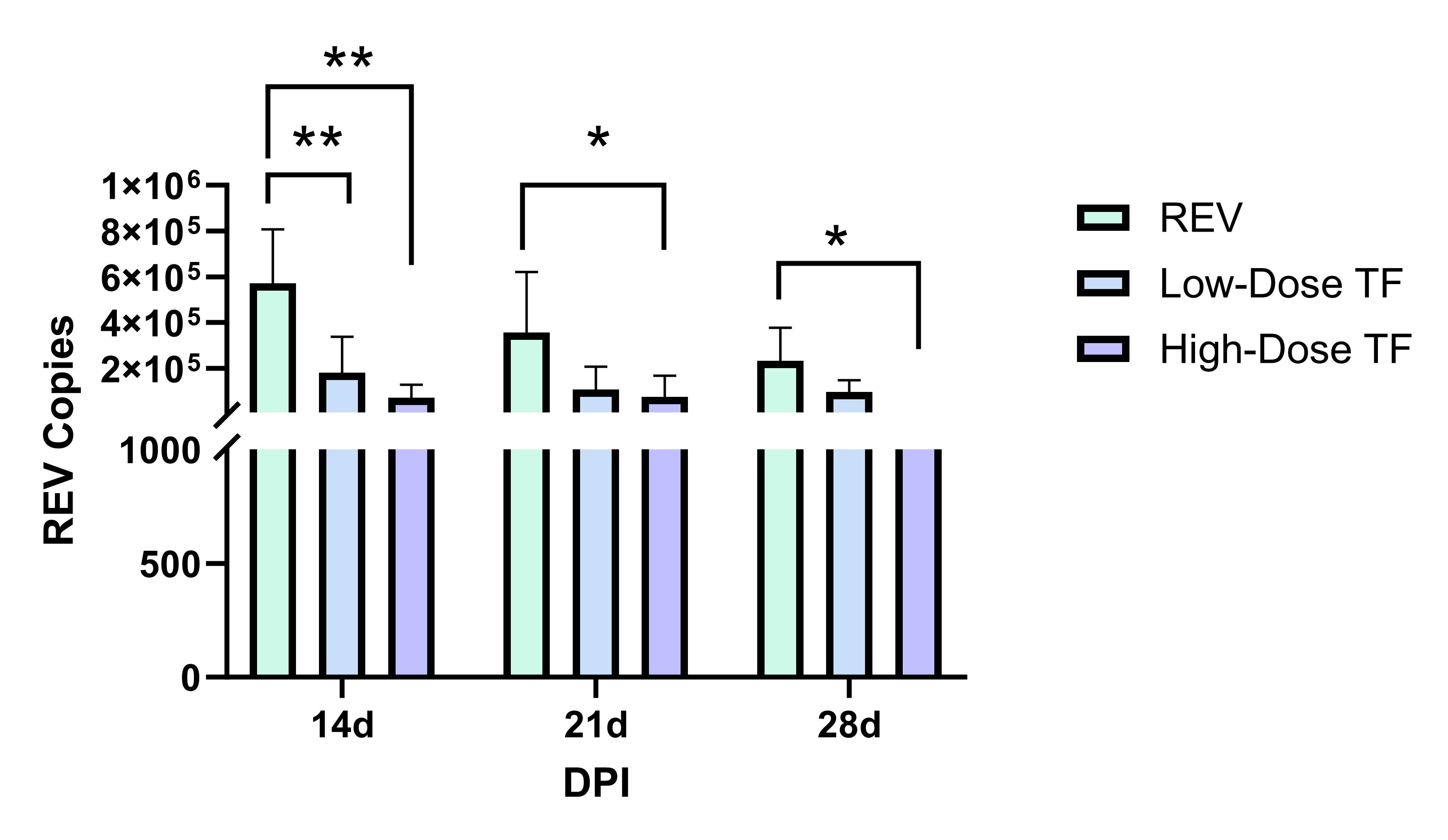

Supplement: Supplementary file 1 [file vetsci-12-01041-s001.zip › figure S4.jpg]

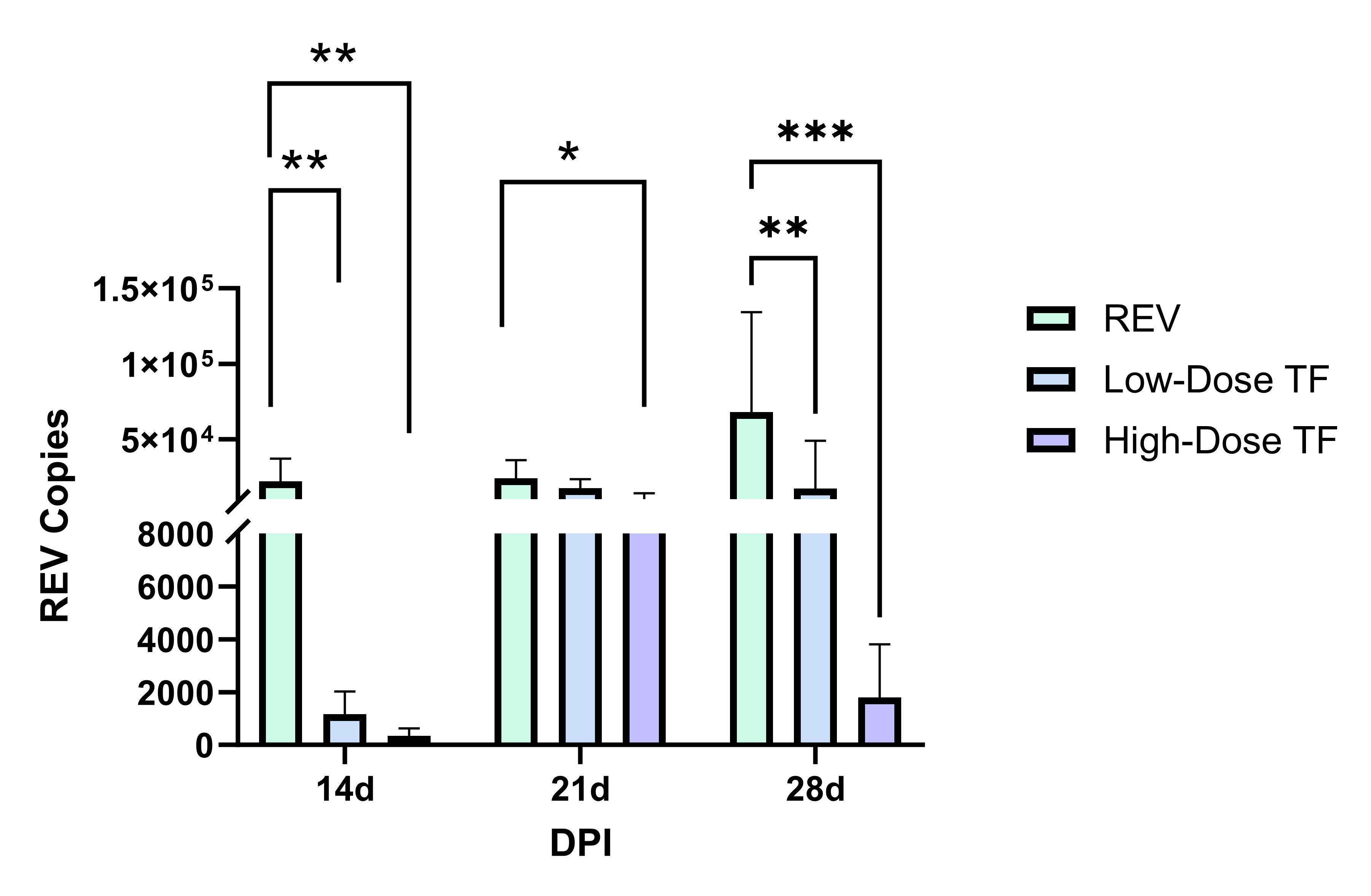

Supplement: Supplementary file 1 [file vetsci-12-01041-s001.zip › figure S5.jpg]

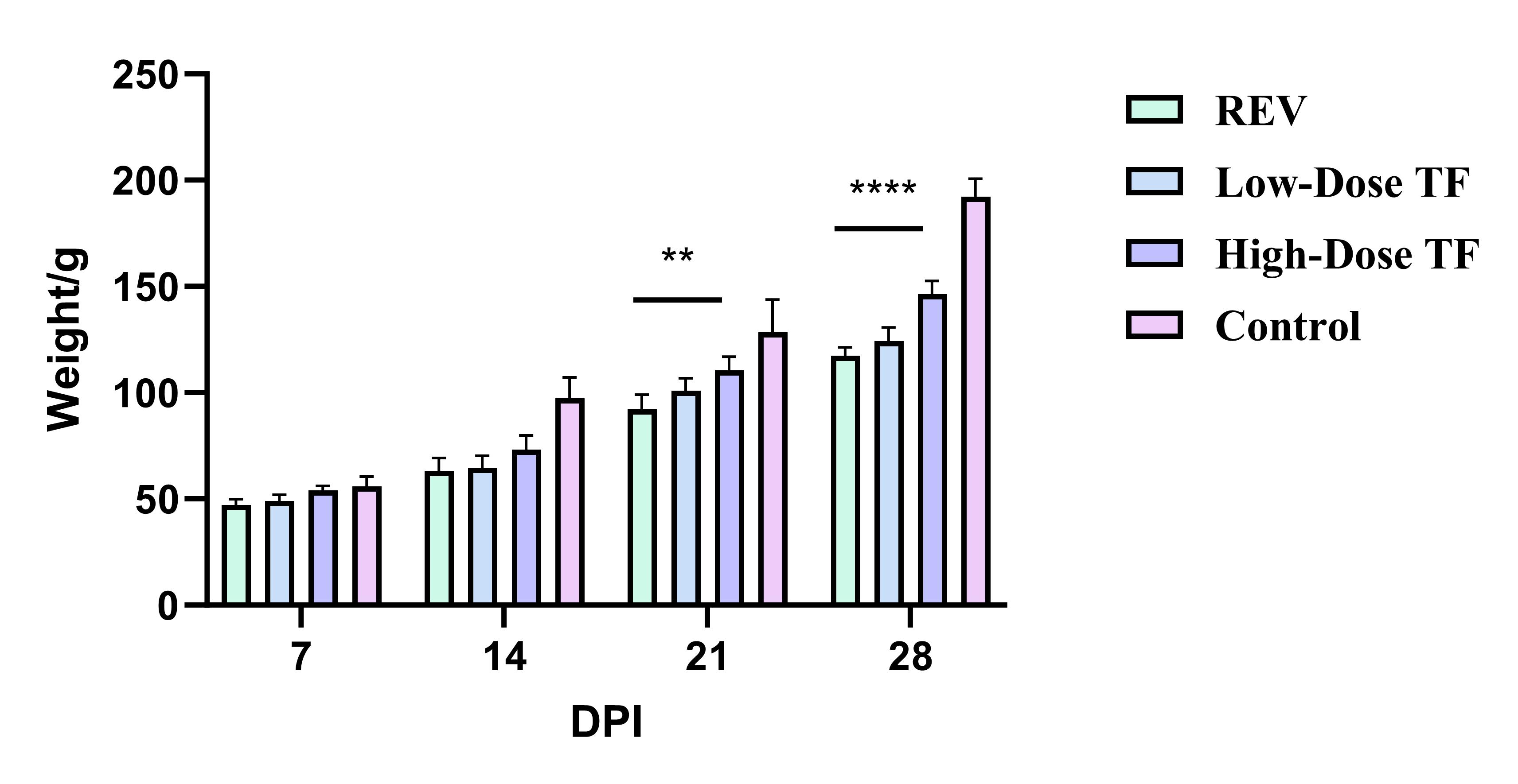

Supplement: Supplementary file 1 [file vetsci-12-01041-s001.zip › figure S6.jpg]

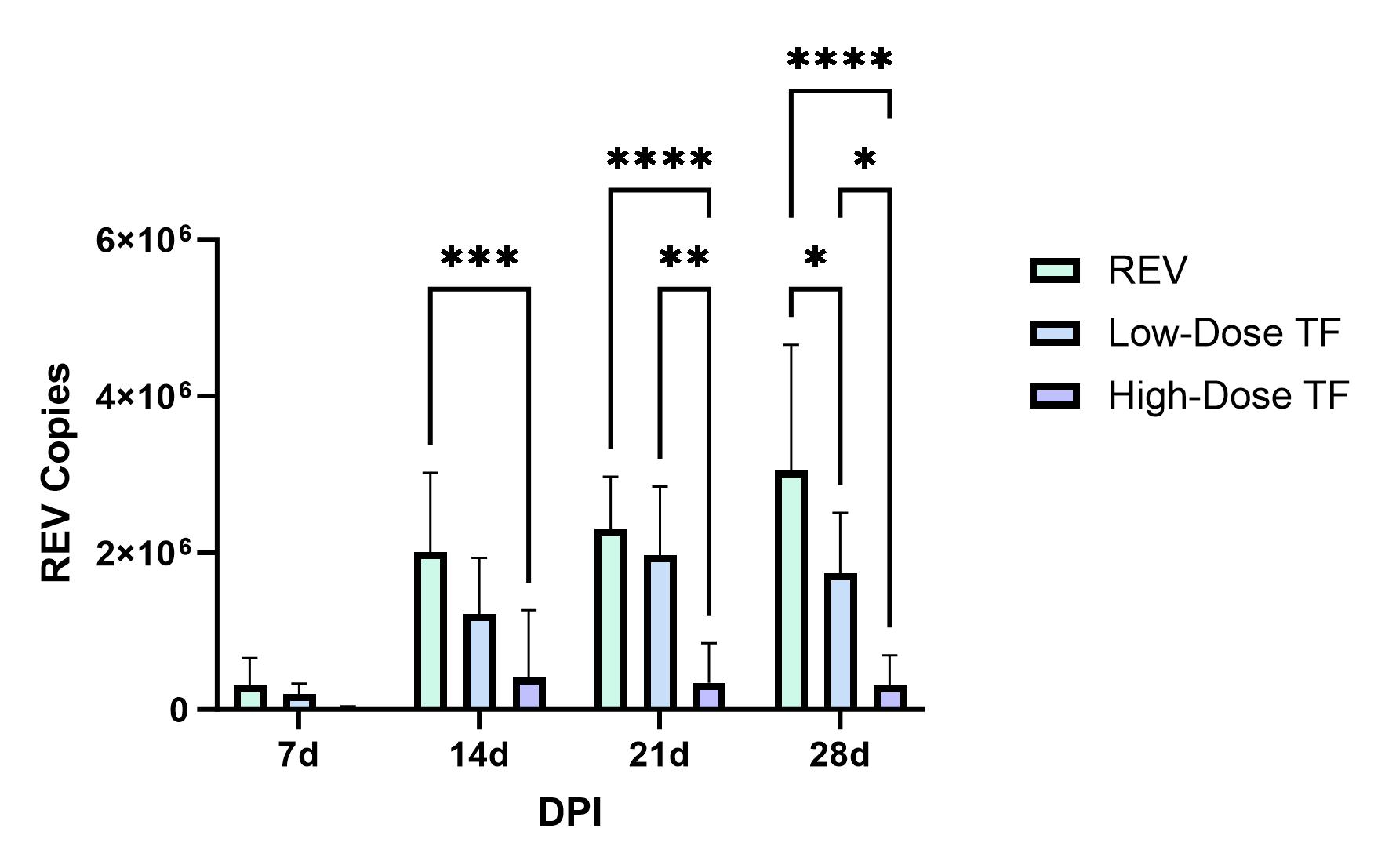

Supplement: Supplementary file 1 [file vetsci-12-01041-s001.zip › figure S7.jpg]

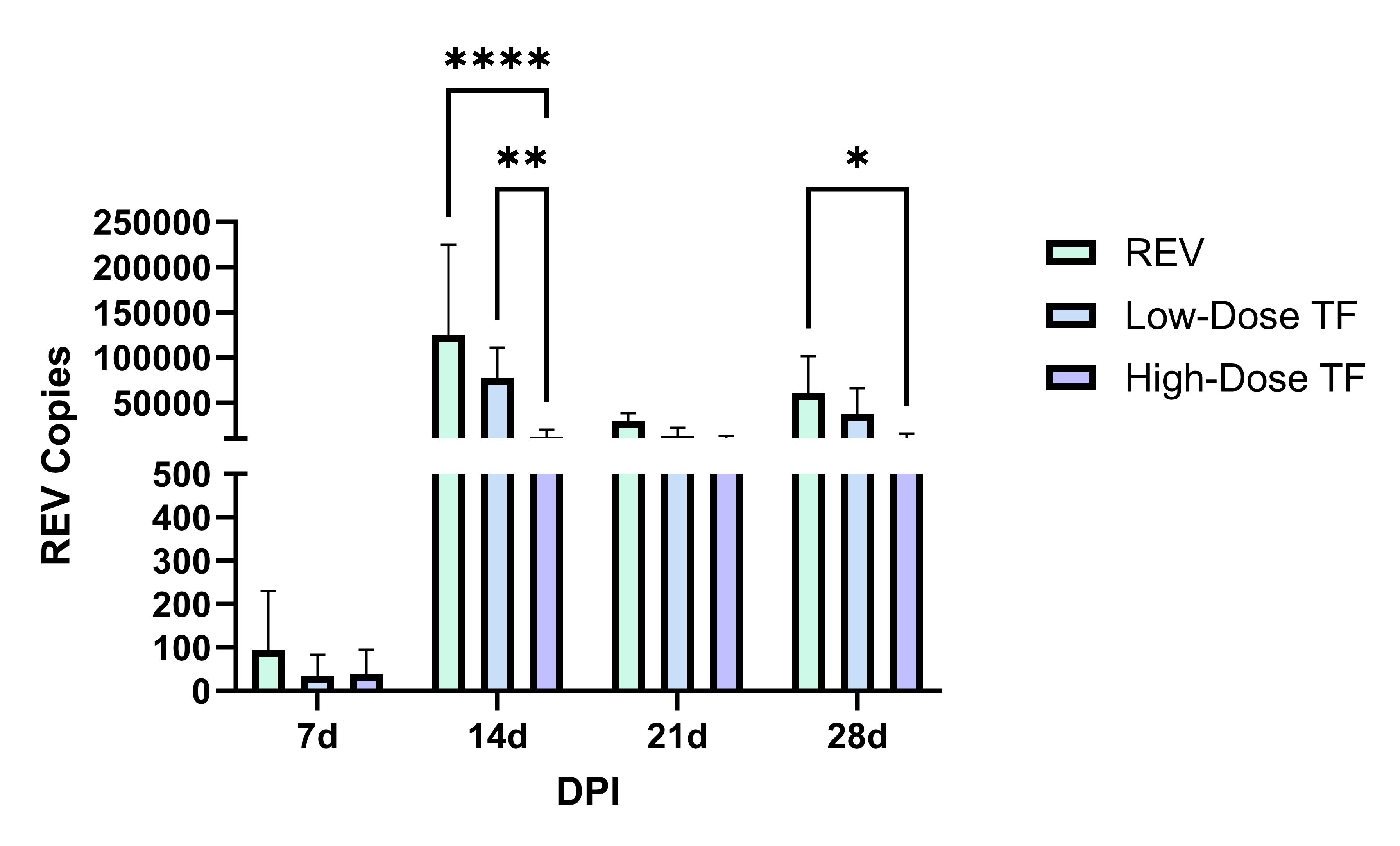

Supplement: Supplementary file 1 [file vetsci-12-01041-s001.zip › figure S8.jpg]

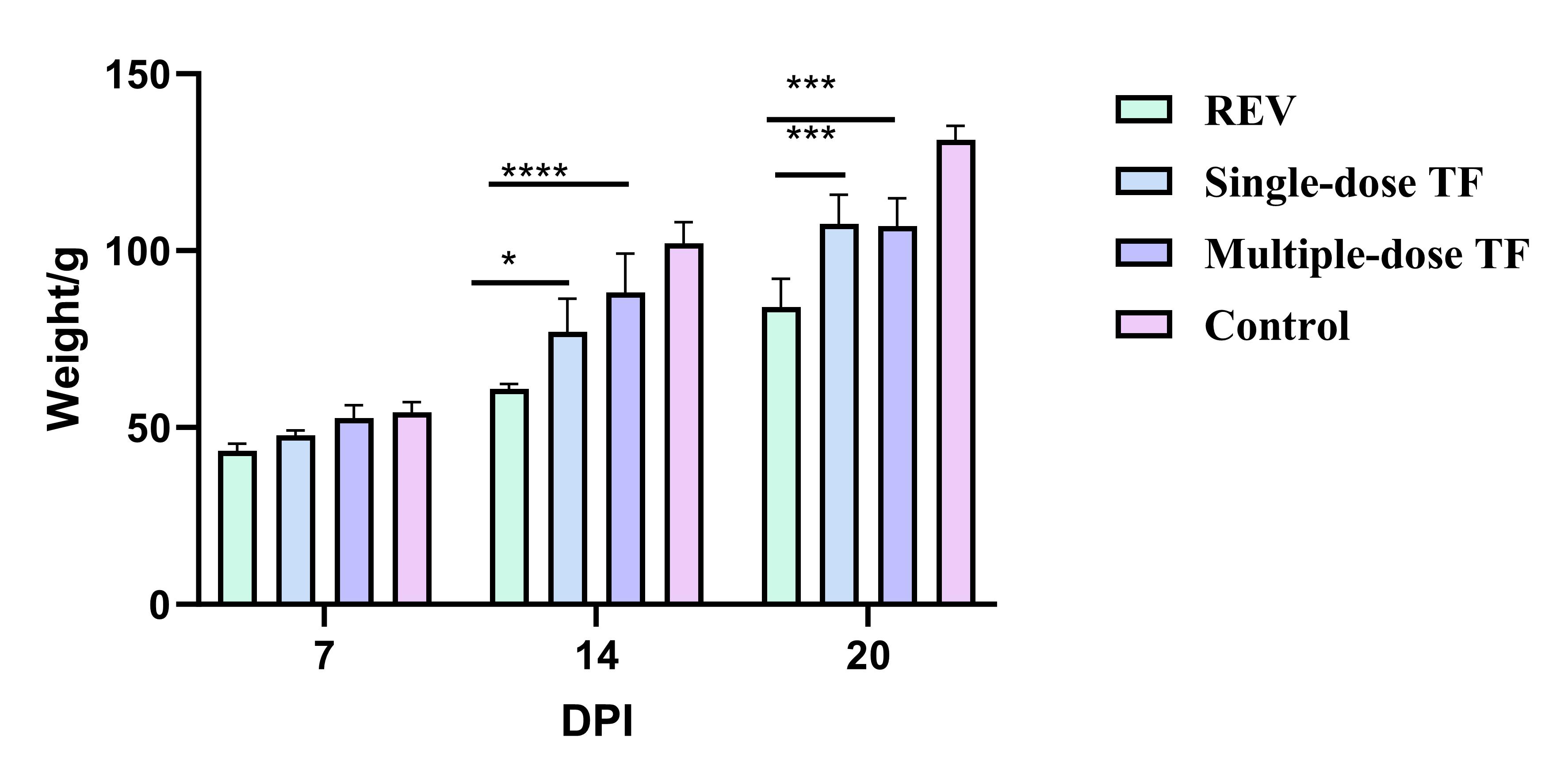

Supplement: Supplementary file 1 [file vetsci-12-01041-s001.zip › figure S9.jpg]
